# Cuba’s cardiovascular risk factors: International comparison of levels and education gradients

**DOI:** 10.1371/journal.pone.0247831

**Published:** 2021-03-04

**Authors:** Maria Dieci, Juan J. Llibre-Rodriguez, Daisy Acosta, William H. Dow

**Affiliations:** 1 Berkeley School of Public Health, University of California, Berkeley, CA, United States of America; 2 Facultad de Medicina Finlay—Albarran, Medical University of Havana, Havana, Cuba; 3 Universidad Nacional Pedro Henriquez Ureña, Santo Domingo, Dominican Republic; University of Mississippi Medical Center, UNITED STATES

## Abstract

**Background:**

Cuba’s life expectancy at 79 is third highest in Latin America. Many attribute this to social investments in health and education, but comparative research is sparse, thus we compare Cuba with neighboring Dominican Republic, Costa Rica due to its strong social protections, and the U.S. Given high cardiovascular mortality, we focus on cardiovascular risk factor levels. To assess the role of health care, we distinguish medically amenable biomarkers from behavioral risk factors. To assess the role of Cuba’s focus on equity, we compare education gradients in risk factors.

**Methods:**

We analyze Cuban data from the 10/66 Dementia Research Group baseline survey of urban adults ages 65 plus. Comparison samples are drawn from the Dominican Republic 10/66 survey, the Costa Rican CRELES, and U.S. NHANES. We analyze cross-country levels and education gradients of medically amenable (hypertension, diabetes, hypercholesterolemia, access to health care) and behavioral (smoking, obesity) risk factors,–using sex-stratified weighted means comparisons and age-adjusted logistic regression.

**Results:**

Neither medically amenable nor behavioral risk factors are uniformly better in Cuba than comparison countries. Obesity is lower in Cuba, but male smoking is higher. Hypertension, diabetes, and hypercholesterolemia levels are high in all countries, though Cuba’s are lower than Costa Rica. Hypertension awareness in Cuba is similar to Costa Rica. Cuba has a higher proportion of hypertensives on treatment than Costa Rica, though lower than the U.S. Comparative gradients by education are similarly mixed. For behavioral factors, Cuba shows the strongest gradients (primarily for men) among the countries compared: smoking improves, but obesity worsens with education. Hypertension awareness also improves with education in Cuba, but Cuba shows no significant differences by education in hypertension treatment.

**Conclusion:**

Smoking is comparatively high in Cuba, but obesity is low, and the resulting biomarkers show comparatively mixed patterns. Cuba’s social protections have not eliminated strong educational gradients in behavioral risk factors, but the healthcare system appears to have eliminated disparities such as in hypertension treatment.

## Introduction

Cuba is a middle-income country with health indicators that mirror those of high-income countries, and a rapidly aging population of 11.3 million. Chronic, non-communicable diseases are the leading cause of death in the country, with Cuba’s aging population reporting a high prevalence of risk factors for cardiovascular disease [1]. This is especially noteworthy because of Cuba’s comprehensive national health care system which is free and government-administered, offering extensive coverage of primary, secondary, and tertiary care to all citizens.

This study compares cardiovascular disease (CVD) risk factor patterns among adults over 65 in Cuba with those from a set of nearby comparison countries: Costa Rica, the Dominican Republic, and the U.S. This study provides a snapshot of health outcomes at a critical age, based on the legacy of the historical momentum of the 1950s and 1960s in Cuba. The comparison countries are heterogeneous in their health and social systems, ranging from Costa Rica’s historically strong social sector and low inequality (often argued to be similar to Cuba) to the substantially less equitable systems in the other countries. We build on prior work such as a six-country study using the WHO’s Study on Global Aging and Adult Health Wave 1 data [2], a study comparing education gradients in CVD risk factors in Costa Rica versus the U.S. [3], and other more recent international comparisons [4–6]. We also build on recent comparative analyses across the 10/66 study countries documenting significant socioeconomic status (SES) gradients in mortality in Cuba that are surprisingly similar to those of the Dominican Republic [7].

The current paper advances these international Cuban comparisons through more detailed analyses of levels and gradients of behavioral and medically amenable CVD risk factor prevalence, awareness, and management among urban adults over 65. We seek to answer the following research questions:

1. What are the levels of key behavioral CVD risk factors such as smoking and obesity among older urban Cuban adults, and how do they compare with similar populations in Costa Rica, the Dominican Republic, and the U.S.?
2. What are the levels of key medically amenable CVD risk factor biomarkers, awareness, and management among older urban Cuban adults, and how do they compare with similar populations in Costa Rica, the Dominican Republic, and the U.S.?
3. Are there socioeconomic (as measured by education) gradients in CVD risk factors among older Cuban adults, and how do they differ in comparison countries?
4. Are there socioeconomic (as measured by education) gradients in medically amenable CVD risk factors, awareness, and management among older Cuban adults, and how do they differ in comparison countries?

We test four hypotheses for how Cuba compares on health risk factors relative to these comparison countries. First, for behavioral risk factors (smoking and obesity), levels in Cuba will be *comparable* to the other countries. For medically amenable risk factors (related to hypertension, diabetes, and cholesterol), levels in Cuba will be *lower* than in the Dominican Republic. We hypothesize that they will be *comparable* to Costa Rica, which has a similarly strong social safety net; we hypothesize the comparison with the U.S. to be more mixed, based on insights from prior work comparing Costa Rica and the U.S. along these dimensions [3]. Regarding socioeconomic gradients, we hypothesize that there will be education gradients in behavioral risk factors in all countries, though possibly flatter in Cuba given its strong social protection programs. A related hypothesis is of weaker education gradients in Cuba than in Dominican Republic and the U.S. for medically amenable factors; this hypothesis is based on Cuba’s history of promoting health and educational equity. For this same reason we also hypothesize that Costa Rican gradients may be somewhat similar to Cuba.

We first review the burden of cardiovascular disease in low- and middle-income countries and gradients by education and discuss the features of the Cuban context that make this population particularly unique. We then discuss the choice of comparison countries, overview the data sources and methods used for analysis, and discuss results on health levels and education gradients with a focus on Cuba. We conclude by putting our findings in the broader context of the existing literature comparing CVD risk factors between and within countries.

## Background and context

### Cardiovascular disease burden in LMICs and education gradients

Noncommunicable diseases (NCDs) are the leading cause of death globally, and 80% of the disease burden due to NCDs is concentrated in low- and middle-income countries (LMICs) [8]. Within the category of NCDs, CVD–mainly ischemic heart disease and stroke–kills more people worldwide than any other cause. A study that documents the prevalence of angina, stroke, and their respective risk factors in six low-and middle-income countries finds a variable burden of CVD across all countries, with large variation in risk factors [2]. The CVD burden around the world is exacerbated by population aging and growth, as well as changes in the distribution of key risk factors–namely tobacco use, obesity, unhealthy diet and lack of physical activity, and harmful use of alcohol [9].

These risk factors are linked to both individual choices and structural barriers that exist within a society and are often a cause and consequence of poverty. There is a vast literature that explores the relationship between socioeconomic status, education gradients, and health outcomes. The relationship between socioeconomic status–of which education is one important measure—and health can be seen in health care, environmental exposure, and health behavior, and occurs throughout the distribution, not just at a threshold cut off of extreme deprivation [10]. Lower socioeconomic status is associated with higher mortality, with the largest disparities occurring between the ages of 45–65 [11–13].

Many studies have explored the link between education and health throughout the life course, and most relevant for this paper, in aging populations. While much of this comparative work focuses on the association between educational attainment and mortality [7, 14–16], a growing literature focuses on behavioral and medically-amenable risk factors for CVD. A comparison of education gradients and CVD risk factors in the U.S. and Costa Rica finds that education gradients on risk factors was stronger in the U.S. than in Costa Rica, despite similar levels of mortality [3]. A population-based study in Australia finds that education plays a protective role for several medically amenable risk factors, but that this relationship is lessened when accounting for behavioral risk factors [17]. A multi-country initiative that measured health among older adults in 1999–2000, Salud Bienestar y Envejecimiento (SABE), finds notable education gradients for multiple CVD risk factors such as obesity, diabetes, and healthcare utilization. Interestingly, education gradients in Cuba–one of the sites included–were significantly smaller for all three of these outcomes [18–20]. Taken together, these studies suggest that the relationship between educational attainment and CVD risk is highly context-dependent and non-uniform across behavioral and medically amenable risk factors.

Cuba is a particularly interesting and understudied case to add to this literature, because of its unique history and social context which produced a universal health care system focused on prevention, a growing aging population that came of age in the midst of the educational and social reforms of the Cuban revolution, and a high CVD burden.

### Cuban setting

Cuba underwent tremendous economic and social change throughout the 20^th^ century, the effects of which were felt across the population, impacting the current generation of older Cubans throughout their lives. Cubans in this age cohort were born in the pre-revolutionary period, in a country with high levels of inequality and poverty, and a poor education system. The revolutionary period brought economic centralization, sweeping social reforms, and nationalization of the health and education systems, and its effects still characterize these contemporary Cuban institutions.

#### Cuban health care system for chronic disease

The Cuban government assumes fiscal and administrative responsibility for the health of its citizens and spends a high proportion of its GDP on the universal health care system. Cuba’s free and comprehensive health care program is charged with providing a full range of health care services for older adults (60+) through community based, hospital, and institutional care [21]. Five hundred community-based polyclinics spread across the country are hubs for 20 to 40 neighbourhood-based family doctor-and-nurse offices, which provide health promotion services, and manage uncomplicated conditions including hypertension, hyperlipidemia and diabetes, for an average of 1500 households each. The family doctor program, with professionals embedded in the neighborhood, was instituted in the 1980s [22]. More recent years have seen a greater shift of specialized care to the primary care level. The high density of medical practitioners, and emphasis on prevention may impact disease management throughout the life course, and into old age.

#### Cuban education system

Cuba’s national literacy campaign, launched in 1961, taught 707,212 adults across the country how to read and write, which increased the national literacy rate to 92% from a national rate of 77% (this was much lower in rural areas). The educational campaign emphasized secondary school attainment and resulted in population-wide improvement in education levels [23]. This younger cohort of current 60–74 year-olds, many of whom co-reside with and are caregivers of their aging parents, has an average educational attainment of 11.1 years, with 57% having completed secondary and university studies [23]. Cuba’s emphasis on educational equity and national education campaigns might translate into flatter education gradients for the country’s older population, if either they or their primary caregivers benefitted from these expansive policies.

#### Economic and social changes

Following the collapse of the Soviet Union in 1989, Cuba saw a steep economic decline like the rest of the Soviet bloc. The crisis and loss of the arrangements that had sustained the Cuban economy prior to the Soviet collapse precipitated major policy shifts in Cuba, which in turn had consequences at the individual and social level including a rise in income inequality. In response to a tightened U.S. embargo and blocking of multilateral assistance, as well as the collapse of commerce and a debt crisis, Cuba sought to bring in currency through other routes, primarily tourism which saw a 21% annual increase in gross income from 1990–2003 [24]. These sectoral shifts have affected the social order and widened inequalities. Urban-rural disparities increased, as rural Cubans were less likely to have transnational personal ties or to have opportunities to work in the tourist sector. This time saw a stark effect on health and nutritional outcomes as well. During the economic crisis, energy intake per capita gradually decreased from 2899 kcal (12 180 kJ) to 1863 kcal/d (7820 kJ/d) and the proportion of physically active adults increased from 30% to 67%. These changes affected the whole population and were sustained for almost 5 years [25].

#### A rapidly aging population

Per World Health Organization statistics, Cuban life expectancy at birth in 2016 was 76.8 for men and 81.3 for women. This is similar to the U.S. and Costa Rica, and higher than the Dominican Republic (70.6 for men and 76.7 for women) [26]. High life expectancy is driven by factors inside the health care system, as well as lifestyle factors that exist outside of the medical arena. As with the majority of Latin American and Caribbean countries, chronic diseases are Cuba’s leading cause of death, causing 60% of all deaths, in order of frequency: heart disease, cancer and cerebrovascular diseases [27]. A particularly high prevalence of vascular risk factors and of chronic non-communicable diseases has previously been documented in the Cuban 10/66 Aging and Alzheimer study in Havana and Matanzas. The majority of participants had hypertension (73%), 24.8% had diabetes, 14.1% had ischemic heart disease, and 7.8% had had a stroke; 85% had more than one cardiovascular risk factor. One fifth of those surveyed were current smokers; 7.5% were classified as high-risk drinkers before age 65 and 3.6% were still in this category [1].

By the year 2020, Cuba is projected to be the country in Latin America with the largest population over the age of 60 (25%) [28], an age group which will likely strain the national health care system. It is helpful to compare Cuba’s health care challenges and successes to those of other countries in order to learn from its unique position.

### Comparison countries

In order to put these Cuban health trends in context, it is useful to compare them to those of its neighbors. We compare CVD behavioral and medically amenable risk factor levels of Cuban urban older adults (over 65) to parallel demographic groups in the Dominican Republic (Cuba’s immediate island neighbor), Costa Rica (a well-studied case with a strong social safety net that is often informally compared to Cuba), and the U.S. We also compare the education gradients in risk factors across these countries, although this is complicated by the fact that the education distributions in each of these countries is distinct. There is a tradition of cross-country comparisons of health outcomes by education gradients, and this paper adds to this literature.

Table 1 provides country comparisons from national statistics along several demographic, socioeconomic, and health dimensions between 2000–2006 to provide context for the analysis. This date range was chosen because the data used for the below analysis was collected in this time period. Descriptive statistics are from World Bank World Development Indicators, 2006, unless indicated otherwise in the table. Costa Rica is the smallest country, with 4.4 million people. The United States, with 299.4 million, is the largest. Cuba and the Dominican Republic have 11.3 and 9.6 million inhabitants, respectively. All four countries have large urban populations. Cuba and the United States have larger shares of residents over the age of 65 (11.4% and 12.3%, respectively) than in the Dominican Republic and Costa Rica (5.7% and 5.9%). Spending on health care is highest in the U.S., at 15.9% of GDP, with the remaining three countries spending between 5.4–7.6% of GDP on health care (though Cuban health care spending as a percentage of GDP has increased since then, to 11% in 2014). Health insurance coverage across countries varies widely also: Cuba has effectively 100% coverage, the U.S. and Costa Rica have over 80% coverage, and the Dominican Republic has less than 30% coverage. The Dominican Republic performs worse on other measures of economic development as well: 42% of households are below the national poverty line, and 22% of households do not have indoor plumbing.

**Table 1:** Country descriptive statistics.

	(1)	(2)	(3)	(4)
	Cuba	Costa Rica	Dominican Republic	U.S.
**Demographic Indicators**				
Population size, millions (2006)	11.3	4.4	9.6	299.4
Over age 65, % (2006)	11.4	5.9	5.7	12.3
Living in urban areas, % (2006)	75.4	62.2	67.5	81.1
**Economic and Health Indicators**				
Per capita GDP, current USD (2006)	4669.06	5245.19	3836.49	46437.07
Healthcare spending, % of GDP (2005)	7.6	7.1	5.4	15.9
Life expectancy at birth, total years (2006)	78	79	72	78
Total uninsured, % (2006, 2000 CR, 2004 US)*	0.0	18.2	73.5	15.7
Physicians per 1,000 (2000–2006)	5.9	1.3	1.9	2.3
**Socioeconomic Status Indicators**				
Households below national poverty line, % (2004)	-	23.9	42.2	11
Adult literacy rate, % (2005)	100	95	87	-
Has improved water source, % (2004)	91	97	95	100
Has improved sanitation facilities, % (2004)	98	92	78	100

Descriptive statistics were obtained from the World Bank World Development Indicators (http://wdi.worldbank.org/tables).

* Insurance information was obtained from the 2006 DHS survey in Dominican Republic, 2004 US census, and 2000 Costa Rican census.

These differences in socioeconomic status and investment in the formal health care sector may affect people’s ability to access high quality chronic disease management services, or access counseling and primary care to address some of the early risk factors for CVD such as obesity and smoking. Cuba performs quite well along measures of development: 98% of households have indoor plumbing, 91% have an improved water source, and Cuba has twice the number of physicians per thousand residents compared to the three other countries. Again, these differences in development status speak to the differences across countries in wealth, which links to differences in health behaviors, environmental exposure, and the health care system–all of which influence CVD risk factors, management, and awareness.

## Data and methods

### Surveys

Comparable samples of adults ages 65+ were drawn from each country. We use the 10/66 Dementia Research Group baseline survey for Cuba and the Dominican Republic [29], the Costa Rican Longevity and Healthy Aging Study (CRELES) baseline survey for Costa Rica [30] and the National Health and Nutrition Examination Survey (NHANES) for U.S. data [31, 32].

Data from Cuba and the Dominican Republic are from the 10/66 Dementia Research Group baseline survey (conducted 2003–2005) that sampled adults aged 65 and above. The data in this paper are drawn from a comprehensive baseline survey comprised of a clinical interview; a health, medical history, and lifestyles interview; a cognitive assessment; a physical examination; and an informant interview. Participants were selected from geographically pre-determined catchment areas, which excluded predominantly middle class or professional areas with high-income earners. Data from these countries are harmonized through the use of the same core measures with cross-culturally validated assessments from detailed questionnaires and biomarker measurement (including from fasting blood samples). For more detail on the full 10/66 research program, please see: [29]. In Cuba, our sample is comprised of 2924 adults from the urban areas of Havana and Matanzas. In the Dominican Republic, our sample is comprised of 1992 adults from Santo Domingo. In both countries, weights were constructed by 5-year age group, gender, and location (in Cuba, either Havana or Matanzas; in the Dominican Republic, Santo Domingo) to generalize results to the demographic characteristics of the urban populations of older adults from which the samples were drawn, based on the 2002 census in each country (results were not sensitive to the use of these weights).

Data from Costa Rica are drawn from the baseline wave of the CRELES (2005), a nationally representative, probabilistic random sample of adults aged 60 and over selected from the 2000 census database. This baseline survey was conducted between November 2004 and September 2006. The original CRELES sample was stratified by 5-year age groups with over-sampling of older adults. The subsample analyzed in this paper consisted of the 1066 adults above the age of 65 in greater San Jose, using weights to generalize to the metro population. More details on CRELES sampling can be found in [30, 33].

Data from the U.S. are from the NHANES 2003–2006, a nationally representative survey of non-institutionalized adults residing in the U.S. who were randomly selected using a four-stage sampling design. For more details on NHANES sampling see [34]. Our sample is restricted to adults 65 and over, for a total sample size of 2665. An important caveat is that subnational urban-only comparisons are not possible with the NHANES data, thus the reported U.S. analyses are representative of the entire country, not just urban areas. As a robustness check (not shown) we replicated the smoking analyses with urban subsamples from the Health and Retirement Survey (HRS) data, finding results comparable to the national NHANES results presented below. Although the HRS does not have harmonized measures to conduct similar checks with other risk factors, we note that the U.S. is 81% urban as classified by the U.S. Census Bureau, thus it makes sense that urban-only results would be quite similar to national NHANES results reported here.

Data from CRELES and NHANES were harmonized with the 10/66 data by constructing outcome variables in a comparable way, restricting the sample to adults over the age of 65, and creating common variable definition conventions across countries.

We conduct a complete case analysis in this paper, with a total sample size across all four countries of 8,647 adults over the age of 65. This breaks down to 2,924 observations in Cuba, 1,066 observations in Costa Rica, 1,992 observations in the Dominican Republic and 2,665 observations in the U.S.. There are 58 instances where a respondent is dropped from the analysis because educational attainment is missing. We have conducted a missingness analysis to compare outcomes for those missing observations to outcomes of respondents in our final sample. Respondents excluded from this analysis because of incomplete education data tend to be older, less likely to be obese and diabetic, and less likely to have visited a doctor. Full results of this analysis can be found in S4 Table. Sample sizes by education level, country and gender can be found in S3 Table.

### Key variables

Below, we provide an overview of key variables used in analysis. For details on the survey questions used in each country to construct these variables, please refer to S1 Table. Cutoff points for all outcomes were chosen to be consistent with prior comparative literature that uses 10/66 and CRELES datasets [3, 29, 30].

#### Education

The measure of socioeconomic status used in the analysis is attained level of formal education, given that it is better measured than other socioeconomic constructs, and it is established early in life thus less susceptible to reverse causality biases. To compare education levels we constructed a 3-category low/medium/high education variable in each country, consistent with prior literature (see: [3–5]). As in these prior studies, one set of education attainment year cutoffs is used in middle-income countries and a different set is used in the U.S., attempting to roughly categorize education into terciles in each of the two settings. “Low education” is categorized as less than primary school attainment in Cuba, Dominican Republic and Costa Rica, and less than high school attainment in the U.S. “Middle education” is categorized as primary school attainment in Cuba, Dominican Republic and Costa Rica, and high school attainment in the U.S. “High education” indicates more than primary school attainment in Cuba, Dominican Republic, and Costa Rica, and more than high school attainment in the U.S. In results not shown, we replicated analyses when instead dividing the top middle-income country category into two groups (some/completed secondary school and more than secondary school), and the bottom U.S. category into two groups (less than high school and some high school); sample sizes are insufficient though to gain precise additional insights beyond our preferred 3-level categorization. Given the imprecision of this attempt at a 4-category analysis, further attempting to harmonize education categories to use the same definitions of low/middle/high across the U.S. and other countries is not feasible either (in addition to the fact that NHANES does not provide detailed education years of attainment for those with less than high school education), thus instead our preferred approach follows that laid out by the prior studies cited above.

#### Ever smoked

In 10/66, having ever smoked was assessed by the question ‘Has there ever been a period when you smoked cigarettes, cigars, or a pipe, chewing tobacco or snuff nearly every day?’ In NHANES and CRELES, having ever smoked was assessed by the question ‘Have you smoked more than 100 cigarettes or cigars in your life?’ Because these questions are not directly comparable, the results on “ever smoked” should be interpreted with caution.

#### Current smoking

In 10/66 current smoking status was assessed by the question ‘Do you still use tobacco regularly?’, while in NHANES and CRELES it was assessed by the question ‘Do you smoke now?’

#### Measured central/abdominal obesity

Binary variable coded as 1 if respondent was measured as having obesity, and 0 otherwise. In all countries, we define obesity as having a waist circumference measure greater than or equal to 88 cm among women and greater than or equal to 102 cm among men, consistent with the NCEP/ATPIII definition. Waist circumference was measured at the midaxillary line.

#### Measured high blood pressure

Binary variable coded as 1 if the average of two blood pressure measurements meets the World Health Organization/International Society of Hypertension (WHO-ISH) criteria of systolic blood pressure greater than or equal to 140 mmHg and/or diastolic blood pressure greater than or equal to 90 mmHg. Coded as 0 otherwise. In Costa Rica, Cuba and the Dominican Republic, blood pressure readings were taken twice, spaced at least 10 minutes apart. In Costa Rica, both were sitting blood pressure measures [35]. In the Dominican Republic and Cuba, one was a sitting measure and one was standing [29]. The average of the two readings was used to calculate the high blood pressure threshold variable. In the US, blood pressure readings were taken up to four times, after at least 5 minutes of sitting quietly and resting [36]. The average of all the readings was used to calculate the high blood pressure threshold. In all countries, blood pressure readings were taken at the same sitting, which may result in overestimation.

#### Hypertension prevalence

Binary variable coded as 1 if patient has hypertension, as defined here, and 0 otherwise. All those with either self-reported hypertension (“have you ever been told by a doctor that you have high blood pressure?”) or a blood pressure measurement meeting the WHO-ISH criteria (SBP> = 140 mmHg and/or DBP > = 90 mmHg) were considered to have hypertension. Respondents with both self-reported hypertension and measured hypertension were coded as 1. Both measured and self-reported variables used to construct the prevalence measure are present in all four countries.

#### Hypertension unawareness

Binary variable indicating that respondent reported that a doctor had never informed them that they had hypertension, among those with measured hypertension. Those who self-report that a doctor informed them of having high blood pressure are considered to be aware.

#### Hypertension treatment

Binary variable indicating that respondent self-reported being on medication for hypertension. Measured using the question ‘Are you currently/still on treatment?’ among those with self-reported hypertension.

#### Measured diabetes

Binary variable indicating measured fasting glucose greater than 126 mg/dl. Although referred to as “measured diabetes,” this single measure of fasting glucose is a somewhat imprecise measure of diabetes and is not intended to be used for clinical diagnosis. Blood samples for fasting glucose were taken early in the morning after an overnight fast.

#### Diabetes prevalence

Binary variable coded as 1 if respondent had measured high fasting glucose (> 126 mg/dl) or positive self-reported answer to the question ‘Has a doctor ever told you that have diabetes?’ Coded as 0 otherwise.

#### Diabetes unawareness

Binary variable indicating that respondent reported that a doctor had never informed them that they had diabetes, among those with measured high fasting glucose.

#### Measured cholesterol

Binary variable indicating measured fasting total cholesterol greater than or equal to 240 mg/dl.

#### Access to health care

In 10/66, access was measured by the question ‘Have you had contact with any government run health services (local doctor, hospital doctor or nurse etc.) in the last three months?’ In CRELES, a binary indicator of whether or not participant accessed health care was constructed from the following question: ‘How many appointments or health care visits with a doctor did you have in the last 3 months?’ In NHANES, access was measured by constructing a binary variable from the question ‘During the past 12 months, how many times have you seen a doctor or other health care professional about your health at a doctor’s office, a clinic, hospital emergency room, at home or some other place?’ The U.S. measure could not be easily scaled to a parallel 3-month indicator, thus is useful only for comparison of education gradients but not absolute levels.

### Sample description

Table 2 contains descriptive statistics of the study sample. The average age of respondents in our sample is 74, and approximately 42% are male. Educational attainment in the Cuban sample indicates that 24% did not complete primary school, 32% completed primary school, and 44% completed more than primary school (secondary or tertiary). Costa Rica had a larger share in the middle group: 8% did not complete primary school, 60% completed primary school, and 33% had higher attainment. The Dominican Republic had the lowest levels of education: 70% did not complete primary school, 19% completed primary school, and 11% completed more than primary school. Finally, in the U.S., 29% did not complete high school, 30% obtained a high school degree, and 41% had higher educational attainment. In all countries, males attained higher levels of education than females. Given these different education distributions across the four countries, care must be taken not to over-interpret precise educational gradient differences across the countries.

**Table 2:** Sample descriptive statistics.

	(1)	(2)		(3)		(4)		(5)	(6)		(7)		(8)	
	*Females*							*Males*						
	Cuba	Costa Rica		Dom. Republic		U.S.		Cuba	Costa Rica		Dom. Republic		U.S.	
Sample size	1902	647		1312		1331		1022	419		680		1334	
*Demographics*	** **													
Respondent age	75	74		75		75		74	74		73		74	
Less than primary school education (high school for U.S.) (%)	28	8	**	74	**	30		18	8	**	64	**	28	**
Completed primary school (high school for U.S.) (%)	34	62	**	17	**	34		30	56	**	22	**	26	*
More than primary school education (high school for U.S.) (%)	38	30	**	10	**	36		52	37	**	13	**	47	*
*Health risk factors*														
Has smoked tobacco in his/her lifetime (> 100 cigarettes equivalent) (%)	30	22	**	40	**	41	**	74	64	**	64	**	70	
Currently smokes tobacco regularly (%)	13	4	**	11		9	**	32	12	**	16	**	12	**
Obesity: Waist circumference > = 88 cm (women) and > = 102 cm (men) (%)	49	65	**	63	**	75	**	20	26	*	21		58	**
Measured high blood pressure: systolic > = 140 mm/Hg, diastolic > = 90 mm/Hg (%)	57	67	**	48	**	46	**	56	68	**	50	*	32	**
Hypertension prevalence: measured, or doctor diagnosis (%)	76	83	**	78		76		70	81	**	75	*	67	
Unaware of hypertension: Measured, but no doctor diagnosis (%)	26	29		16	**	19	**	39	43		29	**	14	**
Treating hypertension: Measured, and on medication (%)	88	61	**	88		94	**	84	59	**	75	**	91	**
Diabetes: fasting glucose measure >126 mg/dl (%)	15	21	**	10	**	18		12	18	*	9	*	17	*
Diabetes prevalence: measured, or doctor diagnosis (%)	29	30		19	**	24	*	20	28	**	17		25	
Diabetes: doctor diagnosis (%)	22	21		15	**	18	**	13	21	**	13		18	**
Unaware of diabetes: Measured, but no doctor diagnosis (%)	19	31	**	20		26		33	26		19	**	36	
High cholesterol: total cholesterol measure > = 240 mg/dl (%)	25	38	**	22		20	**	14	22	*	13		10	*
Respondent has seen a doctor in the last three months (1 year if U.S.) (%)	50	79	**	48		97	**	47	72	**	43		95	**

Country-specific means, using sample weights. Levels of significance are from test of equality of means between Cuba and each comparison country (Cuba as reference case). Levels of statistical significance reported at 5% level (*) and 1% level (**). Obtained by weighted regression of each outcome on country dummy, robust clustered standard errors.

### Statistical analysis

Health levels and gradients are analyzed separately, with all analyses stratified by gender. To examine CVD risk factor levels across countries, we estimate pairwise country means comparisons and report p-values for significant differences in levels across countries. To do so, we use linear probability models for each outcome variable with country dummies as independent variables. We include sample p-weights, which have been normalized to 1 to ensure that each country gets weighted equally, and heteroskedasticity-robust standard errors.

To examine health gradients using education dummies for the three groups, we use logistic regression, controlling for age. We report marginal effects on the probability and statistical significance of each level of education as compared to the lowest level of educational attainment within each country. In NHANES, analyses of blood glucose were examined only in the randomly assigned fasting subsample, and remaining anthropometric outcomes were examined only in the randomly assigned subsample that was tested; appropriate subsample weights were applied. In Cuba, Costa Rica, and the Dominican Republic, analyses of blood glucose and cholesterol were examined only in the subsamples with valid fasting blood draws; this excluded 23%, 13%, and 28% of the samples, respectively, thus weights were adjusted to reflect missingness for these variables. All analyses were conducted in STATA 15 (StataCorp, Texas, U.S.). Probability weights were used to adjust statistics to be representative of older adults as described above, accounting for nonresponse in the subsamples, using the *svyset* command and *pw* options in Stata in all regressions. Standard errors correct for clustered sampling at the PSU level in NHANES using the *cluster()* option in all regressions.

## Results

**Fig 1:**
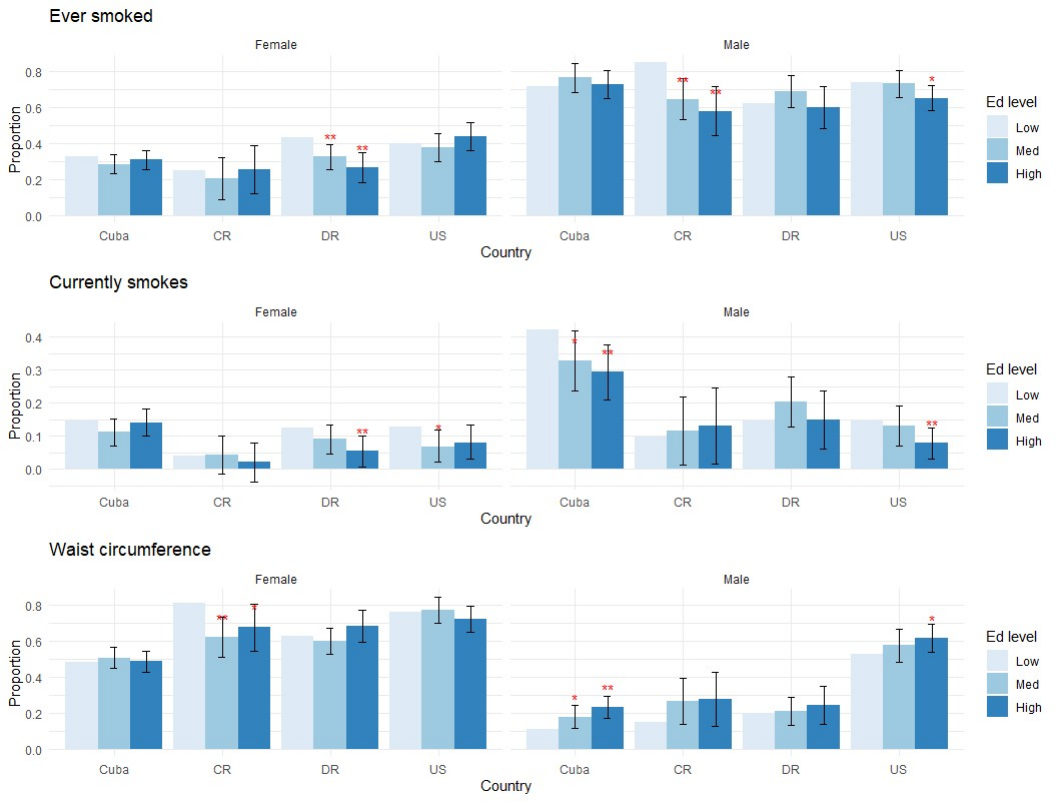
Education gradients: Smoking and obesity. Levels of statistical significance are reported at the 5% level (*) and 1% level (**), comparing middle and high levels of education to the lowest educated group within each country.

**Fig 2:**
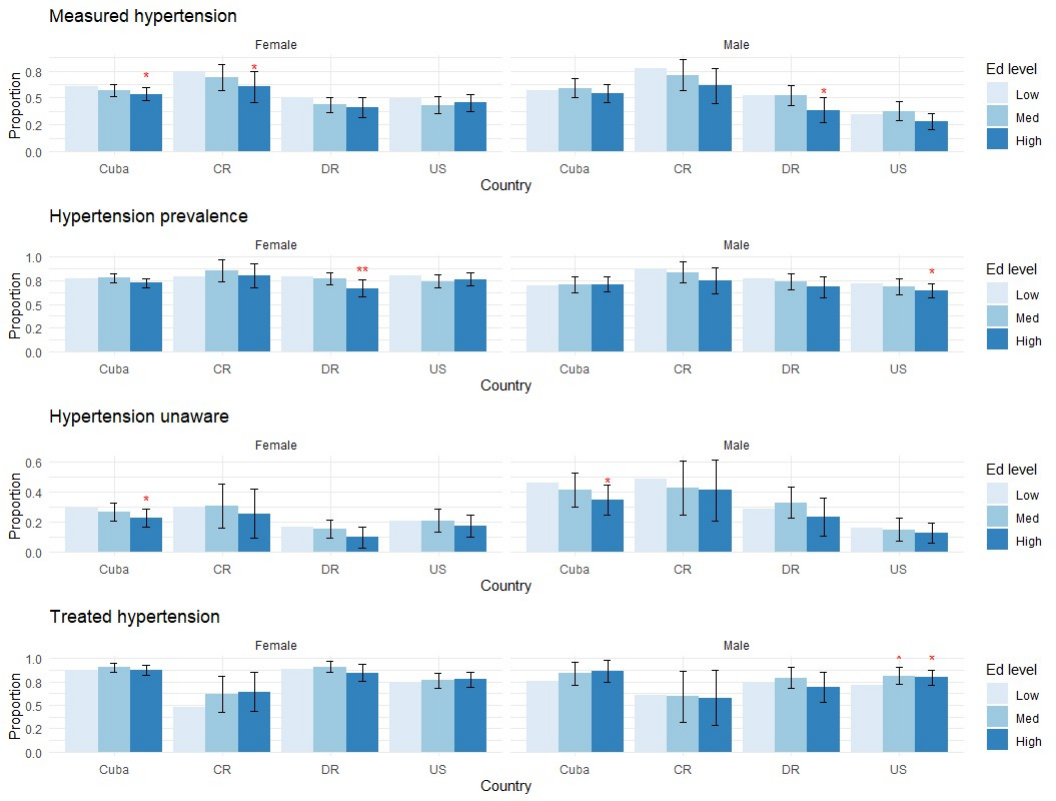
Education gradients: Hypertension. Levels of statistical significance are reported at the 5% level (*) and 1% level (**), comparing middle and high levels of education to the lowest educated group within each country.

**Fig 3:**
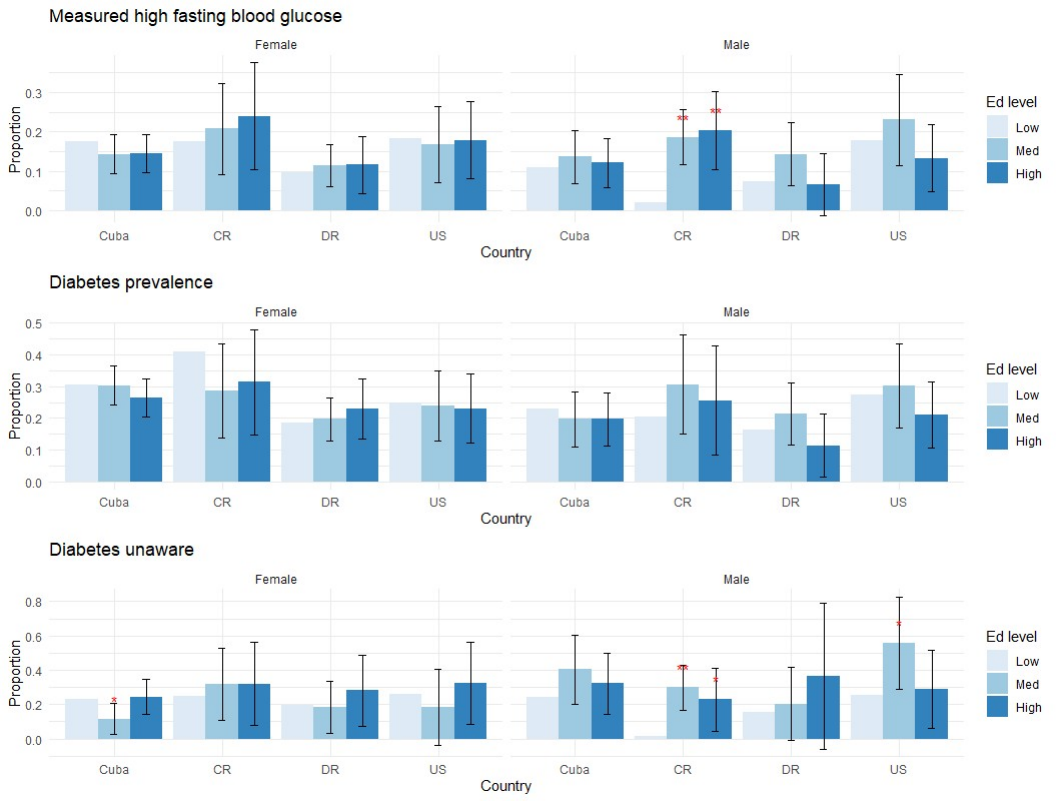
Education gradients: Diabetes. Levels of statistical significance are reported at the 5% level (*) and 1% level (**), comparing middle and high levels of education to the lowest educated group within each country.

**Fig 4:**
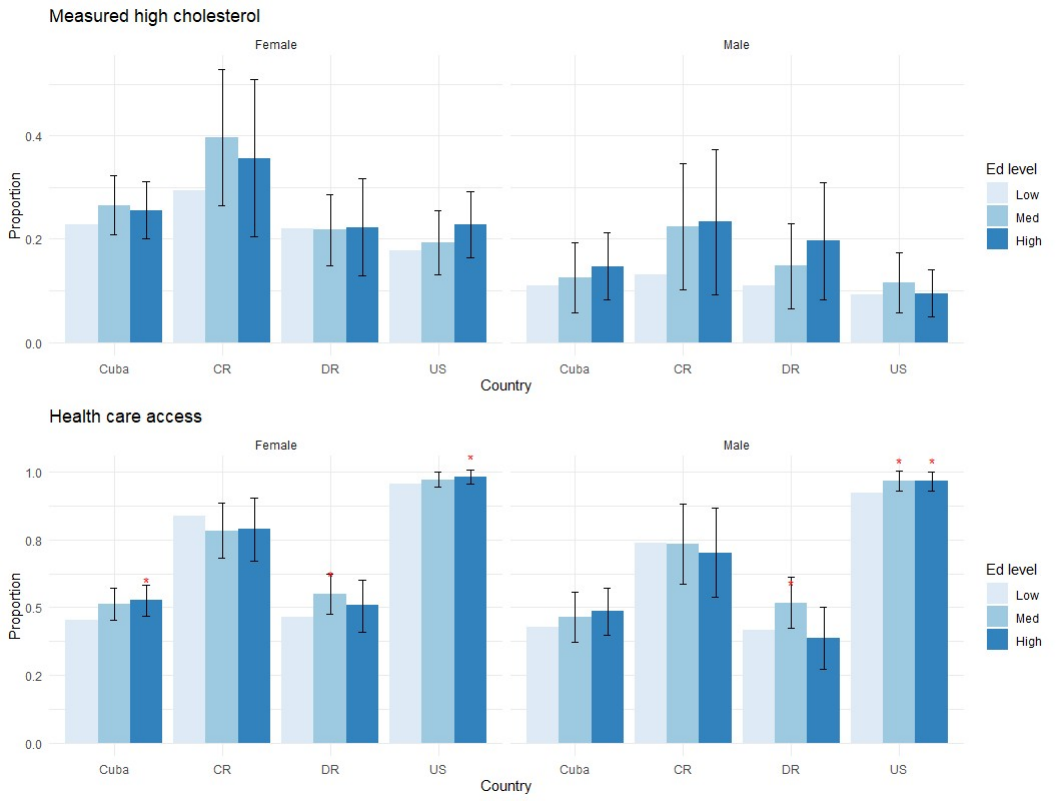
Education gradients: Cholesterol and medical care utilization. Levels of statistical significance are reported at the 5% level (*) and 1% level (**), comparing middle and high levels of education to the lowest educated group within each country.

Table 2, S2 Table and Figs 1–4 present the study findings. We first present behavioral and medically amenable Cuban CVD risk factor levels in comparison to Costa Rica, the Dominican Republic, and the U.S. These results are found in Table 2 and S2 Table. We then discuss how Cuba’s education gradients compare to those of the other three countries for behavioral and medically amenable risks, in turn.

### CVD risk factor levels

Across all four countries, there are notable differences in behavioral CVD risk factor prevalence. Cuban, Dominican and Costa Rican men have a high prevalence of past smoking history, while current smoking prevalence is significantly lower in all three countries with Cuban men still having notably elevated levels. In Table 2, we see the gender breakdown of smoking outcomes by country. Among males, 74% of Cubans have ever smoked, and 32% currently smoke. Among Cuban females, 30% have ever smoked, and 13% currently smoke. In Costa Rica, 64% of males and 22% of females have ever smoked, while in the Dominican Republic 64% of males and 40% of females have ever smoked (all significantly different from Cuba, p<0.01). Looking at current smoking prevalence in these two countries, we see reductions: 12% of men and 4% of women in Costa Rica currently smoke, and 16% of men and 11% of women in the Dominican Republic currently smoke. The burden of smoking in the U.S. appears to be higher historically and lower presently, with 70% of men and 41% of women having ever smoked, and 12% of men and 9% of women currently smoke. These current levels are significantly different from Cuba, comparable to Costa Rica for men, and comparable to the Dominican Republic for women.

Obesity prevalence, another key behavioral risk factor for CVD, differs significantly between men and women within and across countries. In the U.S., 58% of men and 75% of women have high waist circumference, which is the highest prevalence observed across all four countries. In all four countries, women have higher levels of abdominal obesity than men, a contrast to what was found for smoking levels. In Cuba, 20% of men and 49% of women have high waist circumference, with these lower numbers for men partially offsetting the high smoking levels. Cuban men have modestly lower obesity prevalence than Costa Rican and Dominican men, with 20%, 26% (p = 0.029), and 21% (p = 0.670) respectively being considered obese–though the obesity difference between Cuban and Dominican men is not significant. Cuban females have high levels of obesity, but they are much lower (49%) than their counterparts in Costa Rica (65%), the Dominican Republic (63%), and the U.S.(75%); Cuban obesity prevalence for women differs significantly (p<0.01) from the three comparison countries.

The various behavioral risk factors combine to produce hypertension levels that are elevated in all countries. In Cuba, Costa Rica and the Dominican Republic, men and women have a similar burden. Cuban men have measured hypertension levels of 56% and women have measured levels of 57%. Costa Rica, by comparison, has higher levels of measured hypertension: men at 68% and women at 67%. Men in the Dominican Republic have measured levels of 50%, and women have measured levels of 48%. The U.S. has significantly lower levels of measured hypertension, particularly for men: with men at 32% and women at 46%. All countries’ hypertension levels are significantly different from each other, except for women in the Dominican Republic and the U.S.

Turning to look at hypertension awareness and management, we see more differences across countries. Hypertension awareness in Cuba is comparable with that in Costa Rica, significantly lower than in the Dominican Republic, and significantly higher than in the U.S. Thirty nine percent of Cuban men and 26% of Cuban women are unaware of their status, compared to 43% of Costa Rican men and 29% of Costa Rican women. In the Dominican Republic, 29% of men and 16% of women are unaware, and 14% and 19% of U.S. men and women are unaware. Hypertension treatment is high in Cuba for both genders, with 84% of men and 88% of women being on treatment at the time of survey. Treatment in the U.S. is higher, with 91% of men and 94% of women being treated, and treatment among Dominican women is the same as it is in Cuba. Costa Rica has significantly lower treatment levels, with 59% of men and 61% of women being on treatment. All differences across countries for hypertension indicators are statistically significant, except for awareness in Cuba and in Costa Rica, which is comparable for both genders.

Diabetes and hypercholesterolemia levels differ substantially across countries and by gender. The U.S. and Costa Rica have similar levels of measured diabetes, as defined by high fasting blood glucose: 17% and 18% for men, and 18% and 21% for women, respectively. These are higher than in Cuba, where 12% of men and 15% of women have diabetes (though difference between Cuban and U.S. women is not statistically significant), and the Dominican Republic, where 9% of men and 10% of women have diabetes. Awareness of diabetes status has mixed patterns across countries: 33% of Cuban men and 19% of Cuban women are unaware. Costa Rican women are the only group that has significantly higher unawareness of their diabetes status, at 31% (p = 0.009). Dominican men have significantly lower unawareness than Cuban men (19%, p = 0.021), while the other three countries’ men exhibit statistically similar levels.

Cubans have hypercholesterolemia prevalence that is lower than in Costa Rica and higher than in the U.S., for both males and females. Fourteen percent of Cuban males and 25% of Cuban females have measured high cholesterol, as compared to 22% (p = 0.016) of Costa Rican men and 38% (p<0.01) of Costa Rican women, and 10% of U.S. men (p = 0.033) and 20% (p = 0.005) of U.S. women. Dominican hypercholesterolemia levels for both genders look similar to those in Cuba (males: 13% (p = 0.775), females: 22% (p = 0.065)).

Health care access is comparable between Cuba and the Dominican Republic, with about half of respondents having visited a health clinic within the last three months. In Costa Rica, 72% of men (p<0.01) and 79% of women (p<0.01) visited a health clinic within the last three months. Health care utilization is close to 100% in the U.S. for both genders, but visits were measured over the course of one year, so these level differences are not easily interpreted.

### Education gradients

We report the results on education gradients for all outcome variables, grouped by risk factor, in Figs 1–4. Each figure has two panels, one for males and one for females. Each panel displays predicted probabilities for each education level by country. Levels of statistical significance are comparing the predicted probabilities of higher education levels (medium, high) to the low education category within each country.

We find notable education gradients across countries and by gender across our outcome variables of interest. Lower education is associated with a higher likelihood of smoking in the Dominican Republic, Cuba and the U.S. Findings in Fig 1 indicate comparatively high levels of current smoking in Cuba, particularly among men, with a strong negative education gradient: there is more than a 0.4 predicted probability that low educated men currently smoke, compared to just under 0.30 predicted probability of those with high education (p<0.01 versus low educated), with prevalence in Cuba more than double that of men in the other countries. Among Cuban females, however smoking levels are much lower with no strong education gradient.

Higher education is associated with higher likelihood of having obesity in Cuba and the U.S. Low educated Cuban men having a particularly low predicted probability of obesity, of approximately 0.1, with highly educated men having significantly higher predicted probability of obesity (p<0.01). There are no strong education gradients for measured obesity among men in the other countries except the U.S., where the highest educated males have higher probability of obesity than those without a high school degree (p<0.05). Cuban females do not have education gradients for obesity measures, which is consistent with findings from the U.S. and the Dominican Republic, while more educated Costa Rican women have a lower likelihood of having obesity (p<0.05), the only group for which there is a positive education gradient in this measure.

In all countries, lower education is associated with being hypertensive, and in Cuba, Costa Rica, and the Dominican Republic, it is associated with being less aware of hypertension status. The strong negative smoking gradients and positive obesity gradients among Cuba men appear to counterbalance each other to result in no net education gradient in measured hypertension, in contrast to the other countries which all have negative education gradients in measured hypertension among men. Among Cuban women there is a significant negative education gradient in measured hypertension, though it is slightly less marked than in Costa Rica.

More relevant to the health care system are levels of hypertension awareness and treatment among those who are hypertensive: Fig 2 shows that positive education gradients in awareness are at least as strong, if not stronger, in Cuba as compared to other countries. Despite that, in the Cuban healthcare system there are no significant disparities in *treated* hypertension. Costa Rica and Dominican Republic similarly show no gradients in treated hypertension. The sole significant gradient in treated hypertension is among U.S. males, though Cuban confidence intervals overlap the size of this U.S. gradient.

Higher education is also associated with having diabetes in Costa Rica and being unaware of diabetes status in the U.S. and Costa Rica. Measured diabetes, which is affected by the positive education gradient in obesity, shows no net education gradients in Cuba, in contrast to the strong positive gradient in Costa Rica (Fig 3). Diabetes awareness displays U-shaped educational patterns in several groups, which may partly reflect larger confidence intervals given the smaller sample size when restricted to those with diabetes.

Higher education is also linked to increased access to health care services in Cuba, the Dominican Republic and the U.S., results of which are reported in Fig 4. It is much more likely for high educated Cuban women to have used medical services in the past 3 months than low educated women, a gradient that again appears at least as large if not larger than in the comparison countries. Among men there is also a positive gradient in medical service utilization, but it is not statistically significant.

## Discussion

Taken together, these results show that chronic disease risk factors among older urban Cuban adults are not uniformly better than their counterparts in comparison countries, even for medically amenable risk factors. This finding counters our originally stated hypothesis, that medically amenable risk factor levels in Cuba would be similar to those in Costa Rica, and significantly lower than in the Dominican Republic. Although Cuba performs well on some measures such as female obesity and male hypertension treatment, in general high CVD risk factor levels are a major challenge to Cuba’s health care system. Similar to other countries, Cuba will need to continue expanding efforts to adapt its health system to address the rapidly growing non-communicable disease burden. It is encouraging that one of the most medically amenable risk factors, the proportion of hypertensives on medication, is indeed quite high in Cuba and almost as high as in the United States. It is concerning though that despite the strong primary care in Cuba, disease unawareness is still fairly high and generally no better than in the comparison countries.

Furthermore, although some education gradients appear flatter in Cuba, they do still exist. As one summary comparison measure, Cuba has statistically significant (at the 5% level) education gradients for 7/24 (29%) gender-specific outcomes compared. Costa Rica has significant gradients for 5/24 (21%) outcomes. Dominican Republic has gradients for 6/24 (25%) outcomes, and the U.S. has gradients for 9/24 (38%) outcomes. By this metric, and contrary to the original hypothesis, Cuba does not have weaker overall education gradients than in the comparison countries. This finding is particularly interesting given Cuba’s emphasis on equity and a universal free health care system and underscores the importance of factors outside the health care system that influence health behaviors and outcomes. A particular ongoing challenge for Cuba’s health care and public health sector is to reduce current smoking levels, which among low educated men were still well above other groups at 40% at the time of this baseline survey. These results are consistent with a study of smoking and SES in Cienfuegos, which found higher rates of smoking among black and mestizo persons (29.5% vs 25% in the overall population) and the lowest smoking prevalence was among the university educated, at just 16.2% [37].

We acknowledge several limitations of this analysis. We are unable to parse explanations of the factors underlying the observed education gradients–such as the extent to which they may reflect supply-side barriers, demand factors such as lower knowledge among the less educated, or perhaps more recent factors due to rising inequality in Cuba’s Special Period. Analysis of subsequent longitudinal follow-ups will be important for monitoring changing socioeconomic gradients in Cuba. Previous research on Cuba from the SABE study among urban adults aged 60 and over, collected in 1999–2000 five years prior to the data reported here, found no educational gradient in obesity [19]. It is unclear whether the significant gradients found among males in our analysis differed from the earlier work because of rapid changes in obesity, or because of differences in measurement or lack of gender stratification in the earlier work. Future data collection efforts will also be helpful for understanding the generalizability of these results to the full country (beyond the two urban areas of Cuba analyzed here) and to ensure robustness of comparisons across different urbanicity definitions in each country (as noted above, a limitation of the U.S. comparison is use of national NHANES data rather than a metro sample matching urban Cuba). Future data with an even richer array of well-measured health indicators would also be valuable. Larger samples would also enable investigation of alternative socioeconomic categorizations, given the limitations in comparing the somewhat different educational distributions across countries in the present analysis. Additionally, the use of self-reported measures for hypertension and diabetes physician-diagnosed prevalence is a limitation, though consistent with other papers in this literature.

To conclude, this paper adds to the literature on the determinants of CVD risk factors between and within aging populations, by studying Cuba, Costa Rica, Dominican Republic, and the U.S. Cuba, a country that is often considered a leader in universal health care access, is an important addition to these cross-country comparisons. We compare levels and education gradients for key CVD risk factors and find that Cuba does not outperform the comparison countries on either risk factor levels or education gradients, contrary to our original hypothesis. We instead find a more nuanced picture that reflects the complex and contextual nature of determinants of CVD. Smoking is comparatively high in Cuba, but obesity is low, and the resulting biomarkers show comparatively mixed patterns. Cuba, like its neighbors, must continue to address the rapidly growing non-communicable disease burden both within and outside of the health care system. Cuba’s social protections have not eliminated strong educational gradients in behavioral risk factors, but the healthcare system does appear to have eliminated some important disparities, as evidenced by the lack of educational gradients in hypertension treatment.

## Supporting information

S1 Table Variable descriptions. (XLSX)

S2 Table Education and risk factor levels, differences across all countries. (XLSX)

S3 Table Sample sizes, by education level. (XLSX)

S4 Table Analysis of missing values for educational attainment. (XLSX)
